# Associations of a healthy lifestyle score from childhood to adulthood with subclinical kidney damage in midlife: a population-based cohort study

**DOI:** 10.1186/s12882-021-02627-0

**Published:** 2022-01-03

**Authors:** Conghui Liu, Jing Tian, Matthew D. Jose, Ye He, Terence Dwyer, Alison J. Venn

**Affiliations:** 1 Menzies Institute for Medical Research, University of Tasmania, 17 Liverpool Street, Hobart, Tasmania 7000 Australia; 2 School of Medicine, University of Tasmania, Hobart, Tasmania Australia; 3 The George Institute for Global Health, University of Oxford, Oxford, UK

**Keywords:** Healthy lifestyle, Longitudinal studies, Chronic kidney disease, Child, Adult

## Abstract

**Background:**

The relationships of healthy lifestyle scores (HLS) of various kinds in adulthood with the risk of chronic kidney disease (CKD) have been reported, but little is known about the association of childhood lifestyle with later life CKD. This study examined the relationship of HLS from childhood to adulthood with subclinical kidney damage (SKD) in midlife, a surrogate measure for CKD.

**Methods:**

Data were collected in an Australian population-based cohort study with 33 years follow-up. 750 participants with lifestyle information collected in childhood (ages 10–15 years) and midlife (ages 40–50 years), and measures of kidney function in midlife were included. The HLS was generated from the sum scores of five lifestyle factors (body mass index, smoking, alcohol consumption, physical activity, and diet). Each factor was scored as poor (0 point), intermediate (1 point), or ideal (2 points). Log-binomial regression was used to investigate the relationship of HLS in childhood and from childhood to adulthood with SKD defined as either 1) estimated glomerular filtration rate (eGFR) 30–60 mL/min/1.73m^2^ or 2) eGFR> 60 mL/min/1.73m^2^ with urine albumin-creatinine ratio ≥ 2.5 mg/mmol (males) or 3.5 mg/mmol (females), adjusting for socio-demographic factors and the duration of follow-up.

**Results:**

The average HLS was 6.6 in childhood and 6.5 in midlife, and the prevalence of SKD was 4.9% (*n* = 36). Neither HLS in childhood nor HLS from childhood to adulthood were significantly associated with the risk of SKD in midlife.

**Conclusions:**

A HLS from childhood to adulthood did not predict SKD in this middle-aged, population-based Australian cohort.

**Supplementary Information:**

The online version contains supplementary material available at 10.1186/s12882-021-02627-0.

## Background

Chronic kidney disease (CKD) is one of the major preventable chronic diseases associated with progression to end-stage renal disease, cardiovascular disease (CVD), and increased mortality [1]. It was ranked the 12th leading cause of death and resulted in more than 2.5 million (2.17%) deaths worldwide in 2016 [2, 3]. In Australia, 1.8 million hospitalisations were associated with CKD in 2017–18 [4]. Although an estimated one in ten Australian adults (about 1.7 million) in 2011–12 had biomedical signs of CKD in a national health survey, less than 10% of people were aware that they had it because CKD typically has no symptoms at early stages [4, 5].

The risk factors for CKD can appear from before birth through childhood to adulthood, and include fixed (genetic, aging) and modifiable (environment, lifestyle) factors [6, 7]. Kidney functional plasticity peaks in fetal life and is substantial in infants, children, and even adults, providing the potential to reverse the negative effects of unhealthy lifestyle factors (e.g., obesity, smoking, malnutrition) that threaten kidney development and function [8]. Understanding the pathways leading to the development of CKD through a life-course approach may help identify opportunities for early intervention to reduce the burden of CKD in later life.

Individuals’ lifestyle factors can be complex because risk factors often coexist (e.g., people with obesity are more likely to be physically inactive), thus a healthy lifestyle score (HLS) has been used by researchers to summarise lifestyle factors as a whole. Previous studies have found a negative relationship between a HLS (where a high score indicates a healthier lifestyle) and the risk of CVD [9], metabolic syndrome [10], and hypertension [11]. In the same cohort as the present study, Gall et al. [12] reported that a higher HLS in young adults was associated with a better cardiovascular profile including lower diastolic blood pressure and low-density lipoprotein cholesterol, but it was cross-sectional in design and had no data on kidney disease. Only two population-based studies have longitudinally examined the association of a HLS with the risk of CKD and both reported a negative association [13, 14]. However, these two studies were limited by short duration of follow-up (1 and 5 years) and included middle aged and elderly participants. To the best of our knowledge, no study has reported the association of a HLS from childhood to adulthood with later life CKD risk. Doing so is important as the pattern of individual lifestyle factors may be established early in life [15–18].

Using data from the Childhood Determinants of Adult Health (CDAH) study with 33 years follow-up from childhood to midlife, we aimed to assess the relationship of a HLS generated from five lifestyle factors composed of body mass index (BMI), smoking, alcohol consumption, physical activity (PA), and diet in childhood, and from childhood to adulthood, with subclinical kidney damage (SKD) in midlife. We focused on known lifestyle risk factors for CKD in adulthood that were available in our cohort study in both childhood and adulthood [19, 20]. The classification of risk status was based on recommended guidelines with different thresholds for children and adults where available*.* In this study, we used the term SKD to reflect the absence of clinical evidence of kidney disease in this middle-aged study population with an expected low prevalence of CKD. We hypothesized that the participants with a low HLS in childhood and those with persistently unhealthy HLS from childhood to adulthood would have a higher risk of SKD in midlife.

## Methods

### Participants

**Fig. 1:**
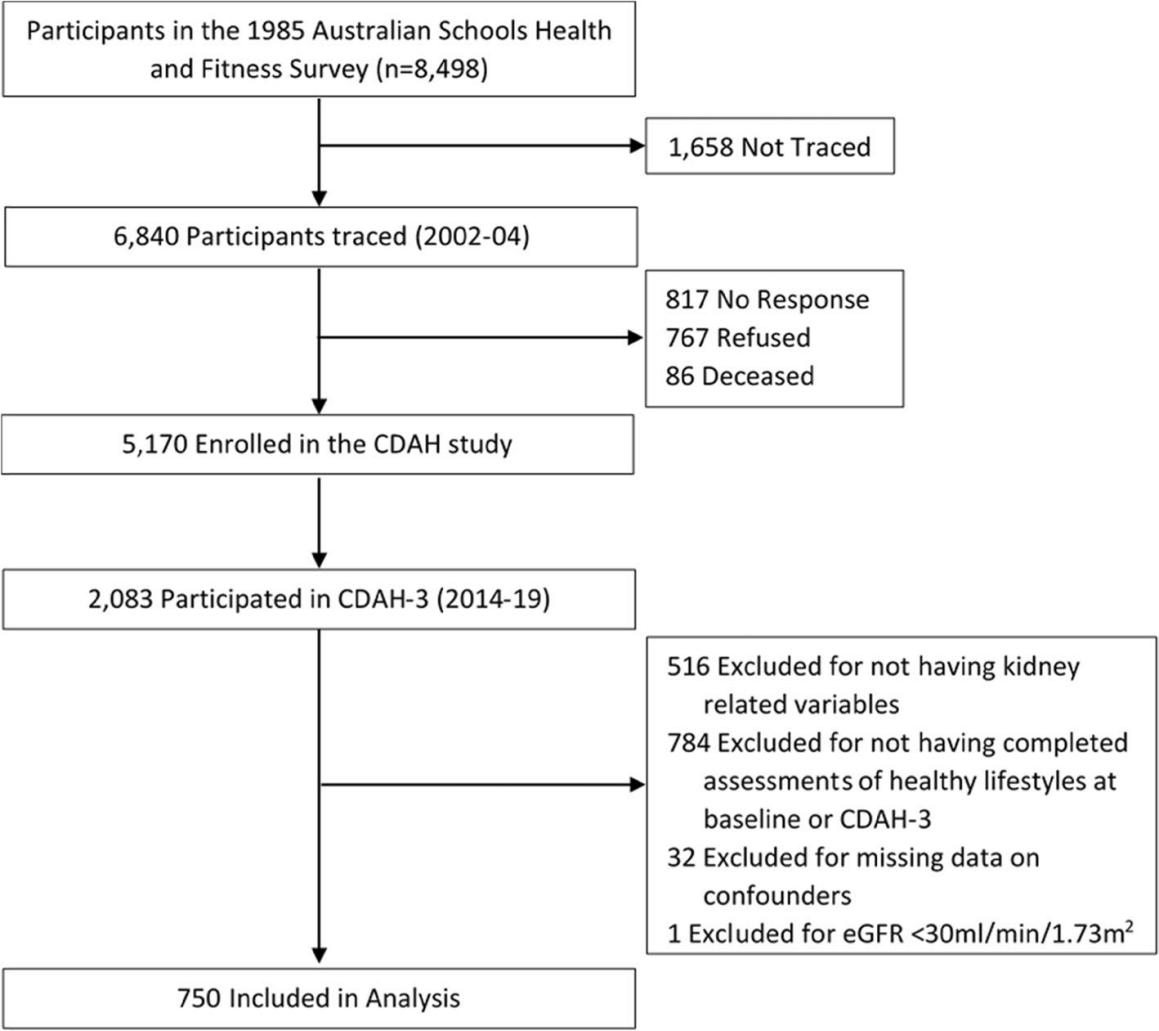
Flow chart of selected participants for Childhood Determinants of Adult Health Study, Australia (CDAH), 1985–2019. eGFR, estimated glomerular filtration rate

This study used data from the CDAH study that began as the 1985 Australian Schools Health and Fitness Survey (ASHFS) with a nationally representative sample of 8498 children aged 7 to 15 years (baseline, also referred to as “childhood”); among them, 6467 children answered questions about their PA, smoking, alcohol consumption, and diet. Details of the study design and extensive measures at baseline including physical (anthropometry, physical fitness), lifestyle and socio-demographic characteristics have been described previously [21, 22]. In 2014–2019, 1566 participants attended a follow-up clinic when aged 36–50 years (midlife); of these, 782 had complete lifestyle information in childhood and midlife (Fig. 1).

At baseline, the Director of Education in each state approved the study and signed parental consent was obtained. At follow-up, participants provided written informed consent, and the study was approved by the Tasmania Health and Medical Human Research Ethics Committee (No. H0013826).

### Lifestyle-related measures

In childhood, BMI (kg/m^2^) was calculated from measured weight and height; overweight and obesity were defined according to international standard age- and sex-specific BMI cut-points [23]. Few participants (*n* = 6) met the criteria for underweight in childhood [24], so children who were not overweight or obese were combined in the same category as “ideal”. Questionnaires were completed by children aged 9–15 years in small groups under the supervision of data collectors who read the instructions to the groups. The children were positioned so that they could not observe the answers given by other children in their group. They were encouraged to ask questions when unsure what was required. Smoking experimentation was defined based on responses to the question “Have you ever smoked even part of a cigarette?”, and children could answer “none”, “a few puffs”, “≤10 cigarettes”, and “> 10 cigarettes”. Alcohol consumption was defined based on responses to the question “How often do you usually drink alcohol?”, and children could answer “never”, “≤once per week”, and those who answered “1-2 days per week”, “3-4 days per week”, “5-6 days per week”, and “everyday” were classified as “>once per week”. Minutes of past-week PA was self-reported and minutes per day derived by dividing by seven. Children were also asked about their effort during PA (“Did you huff and puff?”); those who answered “a lot” were classified as undertaking vigorous PA and “some” as moderate PA. Children aged 10–15 years were invited to participate in the concurrent National Dietary Survey of School children and recorded the time and estimated amount of each food or drink item consumed during a 24-h period; all items consumed were converted to a proportion of a standard serving as defined in the 2013 Australian Dietary Guidelines to score the dietary guideline index (DGI) [25].

In midlife, weight and height were measured, and BMI (kg/m^2^) calculated. Few participants (*n* = 3) met the criteria for underweight in adulthood [26], so adults whose BMI was < 24.9 kg/m^2^ were classified in the same category as “ideal”. Smoking status (never, former, current smoker) was self-reported by questionnaire [27]. Daily alcohol consumption (grams) was calculated based on a Food Frequency Questionnaire (FFQ) [28]. Moderate and vigorous PA were calculated using the long version of the International Physical Activity Questionnaire-long form (IPAQ) [29]. The FFQ was used to assess diet quality using a DGI based on the 2013 Australian Dietary Guidelines for adults [25, 30].

### Healthy lifestyle score

The HLS used in this study comprised five lifestyle factors: BMI, smoking, PA, alcohol consumption and DGI score. Definitions of each HLS component of poor, intermediate, and ideal status for children and adults are shown in Table 1. Each factor was given a point score of zero, one or two to represent poor, intermediate, or ideal status, respectively. No smoking and alcohol consumption were defined as ideal status [12, 31, 32]. The ideal status of PA was defined according to Australia’s Physical Activity and Sedentary Behaviour Guidelines [33]. A total HLS ranging from zero to ten was calculated as the sum of each HLS component scores in childhood and midlife separately. HLS was then dichotomized as unhealthy (score range from 0 to 5), and healthy (6 to 10) categories. HLS from childhood to adulthood was classified as “persistently healthy” (healthy in both childhood and midlife), “improving” (unhealthy in childhood and healthy in midlife), “worsening” (healthy in childhood and unhealthy in midlife), and “persistently unhealthy” (unhealthy in both childhood and midlife) groups.

**Table 1:** Classification of healthy lifestyle factors for children and adults

	Poor (Score = 0)	Intermediate (Score = 1)	Ideal (Score = 2)
**BMI** ^a^			
Children 9–15 years	Obese	Overweight	Normal
Adults> 18 years	≥30.0 kg/m^2^	25.0–29.9 kg/m^2^	< 24.9 kg/m^2^
**Smoking**			
Children 9–15 years	> 10 puffs in my life	A few puffs/< 10 puffs in my life	Never smoke in my life
Adults> 18 years	Current smoker	Former smoker	Never smoke
**Alcohol consumption**			
Children 9–15 years	1–7 days/week	Less than once/week	Never
Adults> 18 years	> 20.0 g/day	0.1–20.0 g/day	0 g/day
**Physical activity**			
Children 9–15 years	0–29.9 min moderate or vigorous activity every day	30.0–59.9 min moderate or vigorous activity every day	≥60.0 min moderate or vigorous activity every day
Adults> 18 years	0–74.9 min/week moderate intensity or 0–37.4 min/week vigorous intensity or 0–74.9 min/week moderate or vigorous intensity	75.0–149.9 min/week moderate intensity or 37.5–74.9 min/week vigorous intensity or 75.0–149.9 min/week moderate or vigorous intensity ^c^	≥150.0 min/week moderate intensity or ≥ 75.0 min/week vigorous intensity or ≥ 150.0 min/week moderate/vigorous intensity
**DGI** ^**b**^			
Children 9–15 years	<25th percentile	25-75th percentile	≥75th percentile
Adults> 18 years	<25th percentile	25-75th percentile	≥75th percentile

^a^In childhood, overweight and obese were defined according to international standard age- and sex-specific body mass index (BMI) cut-points at each age [21]

^b^ The dietary guideline index (DGI) comprises nine indicators reflecting 2013 Australian Dietary guidelines. Seven indicators, worth 10 points each, related to recommended minimum intakes (dietary variety, vegetables, fruit, grains, lean meats and alternatives, low-fat dairy and alternatives, water). Two indicators were for limiting intake of discretionary foods (worth 20 points) and replacing saturated fats with unsaturated fats (10 points). The sum of nine components provides a score out of 100, and a higher score indicates better diet quality [22]

^c^ Minutes of vigorous activity are equal to 2× minutes of moderate activity when moderate and vigorous activities are combined

### Measurement of subclinical kidney damage

Fasting blood and urine samples were collected in the morning and processed according to standardized protocols. Urine albumin was measured by a polyethylene glycol enhanced immunoturbidimetric assay on ADVIA® 2400 Chemistry System analyzer (Siemens Healthcare Diagnostics Inc.; Tarrytown, NY, USA), and creatinine (serum and urine) by the ADVIA Chemistry concentrated Creatinine (CRE_2c) method [34], following the manufacturer’s instructions. Urine albumin-creatinine ratio (UACR) (mg/mmol) was calculated by dividing the urine albumin concentration (milligram/L) by urine creatinine concentration (millimole/L). Creatinine-based estimated glomerular filtration rate (eGFR) was calculated using the CKD epidemiology collaboration (CKD-EPI) formulas [35]. Similar to several other epidemiological studies, SKD was defined as eGFR between 30 and 60 mL/min/1.73m^2^; or eGFR > 60 mL/min/1.73m^2^ with UACR≥2.5 mg/mmol in males or ≥ 3.5 mg/mmol in females [36, 37]. Individuals with eGFR < 30 mL/min/1.73m^2^ were excluded (*n* = 1).

### Covariates

Age and sex in childhood and age in adulthood were collected at the time of enrolment. The duration of follow-up was calculated by subtracting age at baseline from age at follow-up in midlife. Childhood socio-economic position (SEP) (ages 9–15 years) was based on postal code identified from town or suburb of residence reported by ASFHS participants, classified by the socio-economic indexes for areas produced by the Australian Bureau of Statistics [38]. Self-reported health status (very good, good, average, poor, very poor) was collected by questionnaire from children aged 9–15 years. Highest education level achieved in midlife (university education, vocational training, high school) and occupation (manager or professional, white-collar, blue-collar, not in labour force) were collected by questionnaire in adulthood.

### Statistical analyses

Participants’ characteristics in childhood and midlife were presented as means with standard deviations (SD) [or median with interquartile range (IQR)] for continuous variables, and proportions with numbers for categorical variables.

Log-binomial regression models were used to estimate relative risks (RRs) and 95% confidence intervals (CIs) for the associations of HLS in childhood and from childhood to adulthood with SKD in midlife [39]. Multivariable linear regression models were used to examine the association of HLS in childhood and from childhood to adulthood with UACR and eGFR in midlife, independent of socio-demographic factors (childhood age, sex, SEP in childhood, education and occupation in midlife), and the duration of follow-up. Prior to analysis, a Box-Cox transformation was applied to UACR with the purpose of making the residuals more closely normal and less heteroskedastic. The estimated coefficients are reported on the original scale after “back-transformation. The “persistently healthy” group was used as the reference group. Potential confounders were covariates that were independently associated with the outcome, but not mediators (presumed causal consequence of the predictor) between the exposure and outcome, resulting in more than 10% change in the coefficient of the study factor when added into the regression models [40].

Sensitivity analysis was performed to account for loss to follow-up and missing data assumed to be missing at random using inverse probability weighting (IPW) following the approach of Seamen et al. [41]. Three variables with complete data at baseline (age, sex, and school type) were used to impute missing data. SEP, BMI category, waist circumference (WC) z-score, waist to height ratio (WhtR), fitness [sit and reach (cm), sit-ups (number), standing long jump (cm), time for 1.6 km run (minutes)], school enjoyment, school assessed scholastic ability, self-reported health status, smoking experimentation, and passive smoking at baseline were used for the calculation of the weights in IPW.

A two-tailed *P* value of less than 0.05 was considered statistically significant. All analyses were performed using Stata software (Version 16.1, StataCorp, College Station, Texas, USA).

## Results

### Participants’ characteristics

Of the 782 participants with kidney function measurements in midlife and with completed lifestyle information in childhood and midlife, 32 were excluded due to missing data on confounders, leaving 750 participants for the final analyses (Fig. 1). Among those, 740 participants had both UACR and eGFR measures used to define SKD.

The duration of follow-up from childhood to adulthood ranged from 28.8 to 35.9 years, with a mean (SD) length of 33.0 (1.2) years. As shown in Supplemental Table 1, compared with participants who were lost to follow-up, at baseline those included in the study were on average older and fitter, more likely to be female, had higher SEP, better school assessed scholastic ability and self-reported health status, lower BMI and WC, were less likely to have experimented with smoking and had less passive smoke exposure. Compared with the Australian general population of adults aged 35–54 years, a higher percentage of CDAH participants were married or living as married in midlife (73.4% versus 82.5%) [42], employed as professionals or managers (38.0% versus 61.3%) [43], and were never smokers (47.0% versus 64.3%) [44], and a lower proportion were overweight or obese (68.0% versus 62.9%) [45].

The characteristics of participants in childhood and midlife are shown in Table 2. In childhood, the mean (SD) age was 12.5 (1.7) years, and the average HLS was 6.6 (1.5). The largest proportion of participants meeting ideal status for an individual lifestyle factor was for BMI (93.1%) and the smallest was for PA (21.3%). In midlife, the mean age was 45.5 (2.0) years, and the average HLS was 6.5 (1.6). PA had the largest proportion of participants meeting ideal status (85.9%), and alcohol consumption was the factor with the smallest proportion meeting the ideal status (19.1%). 4.9% (36/740) of the total participants had SKD in midlife. Supplemental Table 2 shows the characteristics of the participants in childhood and midlife by HLS from childhood to adulthood. The proportions of participants in each group were: “persistently healthy” (*n* = 456, 60.8%); “improving” (*n* = 105, 14.0%); “worsening” (*n* = 132, 17.6%); and “persistently unhealthy” (*n* = 57, 7.6%).

**Table 2:** Characteristics of participants in childhood (1985) and midlife (2014–19), Childhood Determinants of Adult Health study (*n* = 750)

Characteristics	Childhood	Midlife
Age (year), mean (SD)	12.5 (1.7)	45.5 (2.0)
Males, % (n)	45.2 (339)	–
HLS score, mean (SD)	6.6 (1.5)	6.5 (1.6)
BMI category, % (n)		
Poor	0.8 (6)	24.4 (183)
Intermediate	6.1 (46)	38.5 (289)
Ideal	93.1 (698)	37.1 (278)
Smoking category, % (n)		
Poor	12.8 (96)	9.2 (69)
Intermediate	35.1 (263)	26.5 (199)
Ideal	52.1 (391)	64.3 (482)
Alcohol consumption category, % (n)		
Poor	6.3 (47)	10.3 (77)
Intermediate	26.7 (200)	70.7 (530)
Ideal	67.1 (503)	19.1 (143)
PA category, % (n)		
Poor	58.4 (438)	7.7 (58)
Intermediate	20.3 (152)	6.4 (48)
Ideal	21.3 (160)	85.9 (644)
DGI category, % (n)		
Poor	18.9 (142)	24.7 (185)
Intermediate	53.2 (399)	51.3 (385)
Ideal	27.9 (209)	24.0 (180)
SEP in childhood, % (n)		
High	28.5 (214)	–
Medium-high	28.0 (210)	–
Medium-low	37.2 (279)	–
Low	6.3 (47)	–
Education, % (n)		
University	–	52.7 (395)
Vocational training	–	34.1 (256)
High school or less	–	13.2 (99)
Occupation, % (n)		
Manager or professional	–	61.3 (460)
White-collar	–	18.3 (137)
Blue-collar	–	12.1 (91)
Not in labour force	–	8.3 (62)
Serum creatinine ^a^ (μmol/L), mean (SD)	–	72.6 (15.7)
Urinary creatinine ^a^ (mmol/L), median (IQR)	–	8.7 (9.1)
Urinary albumin ^a^ (mg/L) median (IQR)	–	3.0 (6.0)
UACR (mg/mmol) ^a^, median (IQR)	–	0.5 (0.7)
eGFR (ml/min/1.73m^2^) ^a^, mean (SD)	–	97.7 (12.0)
SKD ^a^, % (n)	–	4.9 (36)

*HLS* healthy lifestyle score; *BMI* body mass index; *PA* physical activity; *DGI* dietary guideline index; *SEP* socio-economic position; *UACR* urinary albumin-creatinine ratio; *eGFR* estimated glomerular filtration rate; *SKD* subclinical kidney damage; *SD* standard deviation; *IQR* interquartile range

^a^ Sample sizes range from 740 to 746

### HLS in childhood and SKD in midlife

No statistically significant associations were found between the HLS as a continuous or categorical variable in childhood and SKD risk in midlife (Table 3). Furthermore, a higher level of UACR in midlife was found in participants with unhealthy HLS in childhood compared with those with healthy HLS (β = 0.09 mg/mmol, 95% CI, 0.01 to 0.18), but this association disappeared after adjustment for socio-demographic factors, and the duration of follow-up. No significant association was found with eGFR in midlife (Table 3).

**Table 3:** The association of childhood HLS as continuous and categorical variables with SKD, UACR, and eGFR in midlife (2014–19)

		SKD RR (95% CI)		UACR (mg/mmol)		eGFR (ml/min/1.73m^**2**^)	
		RR (95% CI)		β 95% CI		β 95% CI	
	SKD/n (%)	Unadjusted	Adjusted ^a^	Unadjusted	Adjusted ^a^	Unadjusted	Adjusted ^**a**^
Childhood HLS	–	1.01 (0.82 to 1.25)	1.04 (0.84 to 1.30)	−0.01 (− 0.03 to 0.01)	−0.01 (− 0.02 to 0.01)	0.04 (− 0.53 to 0.60)	−0.06 (− 0.62 to 0.49)
Childhood HLS category ^b^							
Healthy	27/580 (4.7)	1.00	1.00	1.00	1.00	1.00	1.00
Unhealthy	9/160 (5.6)	1.21 (0.58 to 2.52)	1.07 (0.49 to 2.30)	**0.09 (0.01 to 0.18)**	0.06 (−0.01 to 0.13)	0.26 (−1.85 to 2.36)	0.68 (−1.41 to 2.78)

*HLS* healthy lifestyle score; *SKD* subclinical kidney damage; *UACR* urinary albumin-creatinine ratio; *eGFR* estimated glomerular filtration rate; *RR*relative risk; *CI* confidence interval

^a^ adjusted for childhood age, sex, socio-economic position in childhood, education, occupation in midlife, and the duration of follow-up

^b^ childhood HLS category was defined as unhealthy with HLS range from 0 to 5, and healthy with HLS range from 6 to 10

### HLS groups from childhood to adulthood and SKD in midlife

As shown in Table 4, compared with participants in the “persistently healthy” group, being in other groups did not increase the risk of SKD in midlife. Similarly, no significant differences were found in the associations of HLS groups from childhood to adulthood with the levels of UACR and eGFR in midlife.

**Table 4:** The association of HLS category ^a^ from childhood to adulthood with SKD, UACR and eGFR in midlife (2014–19)

	SKD/n (%)	SKD	UACR (mg/mmol)	eGFR (ml/min/1.73m^**2**^)
		RR (95% CI)	β (95% CI)	β (95% CI)
Persistently healthy	19/449 (4.2)	1.00	1.00	1.00
Improving	5/103 (4.9)	1.00 (0.36 to 2.71)	0.08 (−0.01 to 0.17)	0.12 (−2.43 to 2.66)
Worsening	8/131 (6.1)	1.39 (0.62 to 3.14)	−0.02 (− 0.08 to 0.04)	−1.01 (−3.27 to 1.24)
Persistently unhealthy	4/57 (7.0)	1.46 (0.50 to 4.21)	0.02 (−0.08 to 0.11)	1.03 (−2.19 to 4.26)

*HLS* healthy lifestyle score; *UACR* urinary albumin-creatinine ratio; *eGFR* estimated glomerular filtration rate; *SKD* subclinical kidney damage; *RR* relative risk; *CI* confidence interval

^a^ HLS category was defined as unhealthy with HLS range from 0 to 5, and healthy with HLS range from 6 to 10

Note: adjusted for childhood age, sex, socio-economic position, self-reported health status in childhood, education, occupation in midlife and the duration of follow-up

### Sensitivity analyses

To examine the effects of loss to follow-up on results, data were reanalysed applying IPW. Supplemental Table 3 shows that there are no statistically significant associations between HLS as continuous or categorical variables in childhood and SKD risk in midlife. Although there is no association of HLS in childhood with UACR, the level of eGFR in midlife was 2.38 (0.08 to 4.68) mL/min/1.73m^2^ higher for participants who had unhealthy HLS in childhood compared with those had healthy HLS (Supplemental Table 3). As shown in Supplemental Table 4, compared with participants in the “persistently healthy” group, being in the “worsening” group increased the risk of SKD (RR = 2.32, 95%CI, 1.01–5.31) in midlife. No statistically significant differences were found with the levels of UACR and eGFR in midlife.

## Discussion

This study is the first to report the long-term associations of HLS in childhood, and from childhood to adulthood, with SKD in midlife. We did not find clear evidence of an association in this population-based Australian cohort followed up to age 40–50 years.

The relationship between clusters of healthy lifestyle factors in adulthood and the risk of CKD has been reported previously. A cross-sectional study among 445 men aged 51 years demonstrated that the accumulation of unhealthy lifestyles (lack of habitual or daily exercise; slow walking speed; fast eating speed; late-night dinner; bedtime snacking; skipping breakfast) was associated with a higher prevalence of CKD [46]. Follow-up of participants for 5 years found that changes in habitual exercise and late-night dinner from healthy to unhealthy were associated with an increased incidence of CKD compared with maintaining a healthy status [47]. However, no studies have reported clusters of lifestyle factors in childhood and their associations with CKD in later life. Our study addressed this research gap but found no significant associations between childhood HLS or HLS from childhood to adulthood and the risk of SKD 33 years later. A recent meta-analysis identified 104 studies with more than 2 million adult participants found and reported that the association between modifiable risk factors (diet, physical activity, alcohol consumption, and smoking) and the incidence of CKD was not very strong (less than 25% reduced/increased odds), though the associations were statistically significant [19]. Taking into account previous studies in adulthood [46, 47] and the results from our study, it is possible that the relationship between a HLS and the development of SKD is more apparent with increasing age and greater proximity to the development of CKD.

We found a low prevalence of SKD in this middle-aged (oldest aged 50 years), relatively healthy study sample, which limited the statistical power of the study, but we used continuous UACR and eGFR levels as the outcomes as well. The similarity in results across SKD, UACR and eGFR suggests relative robustness of our findings. The prevalence of CKD would be expected to be higher in older [48] and indigenous Australians [49] and associations with earlier life HLS may be more apparent in these groups. Kidney function slows down with age, and this is accelerated by risk factors including unhealthy lifestyle factors such as obesity and smoking [50]. In Australia, the prevalence of biomedical signs of CKD among people aged 75 years and over (42%) was twice as high as for those aged 65–74 years (21%) and eight times as high as for those aged 18–54 years (6%) in 2011–12 [51]. Thus, our middle-aged participants would be expected to be at an early stage of CKD development consistent with the low prevalence of SKD observed. We found higher levels of eGFR among participants with unhealthy HLS in childhood than in those with a healthy HLS in our study. This is suggestive of hyperfiltration, a physiological response of the kidney to metabolic changes (for example diabetes) that may lead to damaging the nephron [52]. While the progression of CKD is associated with lowering eGFR eventually, previous studies have demonstrated that persons with a decreased complement of nephrons can maintain a normal eGFR initially as individual nephrons enlarge to increase the total surface area available for kidney function (adaptation), which causes glomerular hyperfiltration. This is one of the mechanisms underlying the progression of kidney disease following a variety of kidney injuries [8, 53]. The finding in our study is aligned with our inference that middle-aged participants are at an early stage of CKD development.

There are several additional limitations in our study. First, there was a substantial loss to follow-up, and the subset of participants who participated in study clinics after 33 years may be more health-conscious than those lost to follow-up. We used IPW to account for loss to follow-up and missing data. Although neither association (before and after using IPW) between childhood HLS category and risk of SKD reached statistical significance, there was a marked difference in RRs suggesting that selection bias due to attrition may have reversed the association. Second, we only have a single measurement in childhood, so we were not able to capture change in children’s behaviours over time. Alcohol consumption and smoking were more common in the older participants, but we did not find significant interactions between alcohol consumption or smoking with childhood age. Third, social desirability bias may have occurred since individuals tend to underreport socially undesirable behaviours and over report more desirable behaviours. Measurement error in self-reported behaviour, particularly in childhood, may have led to exposure misclassification. Fourth, although a range of covariates available in the study allowed us to consider multiple potential confounders, residual confounding may still exist due to unmeasured or poorly measured confounders. Further, we only evaluated kidney function in midlife and could not consider the change in kidney function over time as an outcome nor could we adjust for different levels of kidney function at baseline. Finally, we gave the same weights to each lifestyle factor to simplify the process of generating a score, which may not appropriately reflect differentially adverse effects on SKD of the constituent lifestyle factors and prohibits inferences about their possible causal mechanisms. Nevertheless, the approach of equal weighting of different lifestyle factors has worked widely for other diseases associations [9, 12, 54]. Despite these limitations, the present study is the first to investigate the relationship of HLS in childhood and from childhood to adulthood with SKD in midlife. Further research with larger sample size and longer-term follow-up to older ages is necessary to identify the associations between HLS and the risk of CKD.

The major strength of this study is the use of population-based longitudinal study design with 33 years follow-up from childhood to adulthood, allowing us to investigate HLS from childhood to adulthood with early markers of CKD in midlife. This is aligned with the recent emphasis on a “life-course” approach outlined by the World Health Organization for the prevention and control of noncommunicable diseases [55]. Furthermore, the HLS itself is a strength because it is simple to calculate, does not require invasive or lengthy testing, and the items relate directly to modifiable lifestyle factors, rather than biomarkers, which can be translated into goals that are achievable.

## Conclusions

No evidence was found for the association of HLS in childhood and from childhood to adulthood with SKD in this middle-aged and relatively healthy population-based study sample in a developed country.

## Supplementary Information

**Additional file 1.**

## Data Availability

The dataset analysed during the current study are available from the corresponding author on reasonable request.

## Abbreviations

CKD: Chronic kidney disease
CVD: Cardiovascular disease
HLS: Healthy lifestyle score
CDAH: Childhood Determinants of Adult Health
BMI: Body mass index
PA: Physical activity
SKD: Subclinical kidney damage
ASHFS: Australian Schools Health and Fitness Survey
DGI: Dietary guideline index
FFQ: Food Frequency Questionnaire
IPAQ: International Physical Activity Questionnaire-long form
UACR: Urine albumin-creatinine ratio
eGFR: Creatinine-based estimated glomerular filtration rate
CKD-EPI: CKD epidemiology collaboration
SEP: Socio-economic position
SD: Standard deviations
IQR: Interquartile range
RRs: Relative risks
CIs: Confidence intervals
IPW: Inverse probability weighting
WC: Waist circumference
WHtR: Waist to height ratio
