# Supplementary material for: Associations of a healthy lifestyle score from childhood to adulthood with subclinical kidney damage in midlife: a population-based cohort study
Source: BMC Nephrol. 2022 Jan 3;23:2. doi: 10.1186/s12882-021-02627-0 (PMC8722172; doi:10.1186/s12882-021-02627-0)
Supplement: Supplementary file 1 — Additional file 1. [file 12882_2021_2627_MOESM1_ESM.docx]

| **Supplemental Table 1** Baseline characteristics of participants in the study sample and those loss to follow-up | | | |
| --- | --- | --- | --- |
|  | **Include (n=750)** | **Loss to follow-up (n=7748)** | ***P* value** |
| Age (year), mean (SD) | 12.5 (1.7) | 10.8 (2.6) | **<0.001** |
| Males, % (n) | 45.2 (339) | 51.2 (3968) | **0.002** |
| SEP ^*^, % (n) |  |  |  |
| High | 28.5 (214) | 23.0 (1276) | **0.001** |
| Medium-high | 28.0 (210) | 28.7 (1590) |  |
| Medium-low | 37.2 (279) | 38.7 (2148) |  |
| Low | 6.3 (47) | 9.6 (535) |  |
| BMI z score ^*^, median (IQR) | -0.22 (1.11) | -0.16 (1.14) | **<0.001** |
| BMI category ^*^, % (n) |  |  |  |
| Normal | 93.1 (698) | 87.8 (6793) | **<0.001** |
| Overweight/Obese | 6.9 (52) | 12.3 (948) |  |
| WC z score ^*^, median (IQR) | -0.26 (1.04) | -0.15 (1.11) | **<0.001** |
| WHR ^*^, mean (SD) | 0.82 (0.06) | 0.84 (0.06) | **<0.001** |
| WHtR ^*^, mean (SD) | 0.42 (0.04) | 0.44 (0.04) | **<0.001** |
| WHtR category ^*^, % (n) |  |  |  |
| <0.5 | 96.3 (721) | 92.3 (7144) | **<0.001** |
| ≥0.5 | 3.7 (28) | 7.7 (594) |  |
| Sit and reach (cm) ^*^, median (IQR) | 6.0 (10.0) | 4.0 (10.0) | **<0.001** |
| Sit -ups (number) ^*^, mean (SD) | 50.7 (31.3) | 37.2 (29.2) | **<0.001** |
| Standing long jump (cm) ^*^, mean (SD) | 157.0 (25.8) | 141.8 (28.9) | **<0.001** |
| Time for 1.6 km run (minutes) ^*^, mean (SD) | 8.6 (1.6) | 9.3 (2.0) | **<0.001** |
| Smoking experimentation ^*^, % (n) |  |  |  |
| None | 52.1 (391) | 56.1 (3169) | **0.010** |
| A few puffs | 25.2 (189) | 23.1 (1305) |  |
| ≤10 cigarettes | 9.9 (74) | 6.9 (392) |  |
| >10 cigarettes | 12.8 (96) | 13.8 (779) |  |
| Alcohol assumption ^*^, % (n) |  |  |  |
| Never | 67.1 (503) | 67.2 (3793) | 0.156 |
| ≤1 per week | 26.7 (200) | 24.8 (1398) |  |
| >1 per week | 6.3 (47) | 8.1 (456) |  |
| School enjoyment ^*^, % (n) |  |  |  |
| All of the time | 10.1 (76) | 11.2 (636) | **<0.001** |
| Most of the time | 44.3 (332) | 34.3 (1941) |  |
| Some of the time | 37.9 (284) | 39.7 (2244) |  |
| A little of the time | 5.6 (42) | 8.4 (476) |  |
| None of the time | 2.1 (16) | 6.4 (360) |  |
| School assessed scholastic ability ^*^, % (n) |  |  |  |
| Excellent | 15.9 (112) | 8.7 (631) | **<0.001** |
| Above average | 39.4 (278) | 26.6 (1933) |  |
| Average | 34.4 (243) | 41.9 (3036) |  |
| Below average | 9.1 (64) | 17.5 (1272) |  |
| Poor | 1.3 (9) | 5.3 (383) |  |
| Self-reported health status ^*^, % (n) |  |  |  |
| Very good | 40.3 (302) | 34.9 (1974) | **<0.001** |
| Good | 43.3 (325) | 43.6 (2465) |  |
| Average/Poor/Very poor | 16.4 (123) | 21.5 (1218) |  |
| Passive smoking ^*^, % (n) | 37.2 (278) | 51.2 (2877) | **<0.001** |

SD, standard deviation; IQR, interquartile range; SEP, socio-economic position; BMI, body weight index; WC, waist circumference; WHR, waist to hip ratio; WHtR, waist to height ratio; PA, physical activity.
Boldface indicates statistical significance (p<0.05).

^*^ Sample sizes range from 6299-8496 due to missing data at baseline.

**Supplemental Table 2** Characteristics of participants in childhood (1985) and midlife (2014-19) by HLS category ^*^ from childhood to adulthood

| **Characteristics** | **Persistently healthy**  **(n=456)** | **Improving**  **(n=105)** | **Worsening**  **(n=132)** | **Persistently unhealthy**  **(n=57)** | **P value** |
| --- | --- | --- | --- | --- | --- |
| **Childhood** |  |  |  |  |  |
| HLS score, mean (SD) | 7.3 (1.1) | 4.4 (0.8) | 7.1 (0.9) | 4.4 (0.8) | **<0.001** |
| Age (year), mean (SD) | 12.2 (1.7) | 13.3 (1.6) | 12.3 (1.6) | 13.3 (1.5) | **<0.001** |
| Males, % (n) | 45.0 (205) | 37.1 (39) | 49.2 (65) | 52.6 (30) | 0.179 |
| BMI category, % (n) |  |  |  |  |  |
| Obese | 0.2 (1) | 1.9 (2) | 0 | 5.3 (3) | **<0.001** |
| Overweight | 2.6 (12) | 11.4 (12) | 9.1 (12) | 17.5 (10) |  |
| Normal | 97.2 (443) | 86.7 (91) | 90.9 (120) | 77.2 (44) |  |
| BMI z score, median (IQR) | -0.38 (0.98) | -0.09 (1.16) | -0.10 (1.17) | 0.36 (1.06) | **<0.001** |
| Smoking, % (n) |  |  |  |  |  |
| > 10 puffs in my life | 2.2 (10) | 42.9 (45) | 13.6 (18) | 40.4 (23) | **<0.001** |
| A few/< 10 puffs in my life | 32.5 (148) | 43.8 (46) | 30.3 (40) | 50.9 (29) |  |
| Never smoke in my life | 65.4 (298) | 13.3 (14) | 56.1 (74) | 8.8 (5) |  |
| Alcohol consumption, % (n) |  |  |  |  |  |
| 1-7 days/week | 2.4 (11) | 19.1 (20) | 2.3 (3) | 22.8 (13) | **<0.001** |
| Less than once/week | 19.3 (88) | 49.5 (52) | 22.7 (30) | 52.6 (30) |  |
| Never drink | 78.3 (357) | 31.4 (33) | 75.0 (99) | 24.6 (14) |  |
| PA category, % (n) |  |  |  |  |  |
| 0-29.9 min/day | 53.3 (243) | 87.6 (92) | 46.2 (61) | 73.7 (42) | **<0.001** |
| 30.0-59.9 min/day | 21.9 (100) | 10.5 (11) | 22.0 (29) | 21.1 (12) |  |
| ≥ 60.0 min/day | 24.8 (113) | 1.9 (2) | 31.8 (42) | 5.3 (3) |  |
| Vigorous to moderate PA (min/day), median (IQR) | 25.7 (48.9) | 13.6 (21.4) | 32.5 (60.4) | 20.0 (18.6) | **<0.001** |
| DGI category, % (n) |  |  |  |  |  |
| <25th percentile | 11.6 (53) | 45.7 (48) | 12.1 (16) | 43.9 (25) | **<0.001** |
| 25-75th percentile | 54.0 (246) | 46.7 (49) | 56.8 (75) | 50.9 (29) |  |
| ≥ 75th percentile | 34.4 (157) | 7.6 (8) | 31.1 (41) | 5.3 (3) |  |
| DGI, mean (SD) | 48.0 (11.2) | 38.2 (9.4) | 47.9 (11.2) | 37.8 (8.9) | **<0.001** |
| SEP, % (n) |  |  |  |  |  |
| High | 28.3 (129) | 35.2 (37) | 23.5 (31) | 29.8 (17) | **0.032** |
| Medium-high | 28.5 (130) | 22.9 (24) | 31.1 (41) | 26.3 (15) |  |
| Medium-low | 38.8 (177) | 28.6 (30) | 37.9 (50) | 38.6 (22) |  |
| Low | 4.4 (20) | 13.3 (14) | 7.6 (10) | 5.3 (3) |  |
|  |  |  |  |  |  |
| **Midlife** |  |  |  |  |  |
| Age (year), mean (SD) | 45.2 (2.0) | 46.2 (2.1) | 45.4 (1.8) | 46.5 (2.1) | **<0.001** |
| HLS score, mean (SD) | 7.3 (1.1) | 7.1 (1.0) | 4.4 (0.9) | 4.5 (0.8) | **<0.001** |
| Summing HLS score in childhood and midlife, mean (SD) | 14.6 (1.6) | 11.5 (1.3) | 11.5 (1.3) | 8.8 (1.1) | **<0.001** |
| BMI category, % (n) |  |  |  |  |  |
| Obese | 13.6 (62) | 13.3 (14) | 52.3 (69) | 66.7 (38) | **<0.001** |
| Overweight | 38.6 (176) | 50.5 (53) | 36.4 (48) | 21.1 (12) |  |
| Normal | 47.8 (218) | 36.2 (38) | 11.4 (15) | 12.3 (7) |  |
| BMI, mean (SD) | 26.0 (4.7) | 26.6 (4.8) | 30.5 (5.2) | 31.9 (5.5) | **<0.001** |
| Smoking status, % (n) |  |  |  |  |  |
| Current smoker | 2.2 (10) | 8.6 (9) | 26.5 (35) | 26.3 (15) | **<0.001** |
| Former smoker | 21.1 (96) | 29.5 (31) | 35.6 (47) | 43.9 (25) |  |
| Never smoke | 76.8 (350) | 61.9 (65) | 37.9 (50) | 29.8 (17) |  |
| Alcohol consumption, % (n) |  |  |  |  |  |
| > 20.0 g/day | 5.0 (23) | 7.6 (8) | 25.8 (34) | 21.1 (12) | **<0.001** |
| 0.1-20.0 g/day | 73.7 (336) | 67.6 (71) | 64.4 (85) | 66.7 (38) |  |
| 0 g/day | 21.3 (97) | 24.8 (26) | 9.9 (13) | 12.3 (7) |  |
| Alcohol consumption, (g/day), median (IQR) | 4.3 (8.8) | 4.3 (11.1) | 7.3 (18.5) | 7.1 (14.2) | **<0.001** |
| PA category, % (n) |  |  |  |  |  |
| 0-74.9 min/week | 3.1 (14) | 1.9 (2) | 23.5 (31) | 19.3 (11) | **<0.001** |
| 75.0-149.9 min/week | 4.8 (22) | 2.9 (3) | 10.6 (14) | 15.8 (9) |  |
| ≥ 150.0 min/week | 92.1 (420) | 95.2 (100) | 65.9 (87) | 64.9 (37) |  |
| Vigorous to moderate PA (min/week), median (IQR) | 555.0 (606.1) | 560.0 (655.0) | 360.0 (599.4) | 300.0 (552.9) | **<0.001** |
| DGI category, % (n) |  |  |  |  |  |
| <25th percentile | 13.6 (62) | 14.3 (15) | 62.9 (83) | 43.9 (25) | **<0.001** |
| 25-75th percentile | 56.4 (257) | 51.4 (54) | 34.1 (45) | 50.9 (29) |  |
| ≥ 75th percentile | 30.0 (137) | 34.3 (36) | 3.0 (4) | 5.3 (3) |  |
| DGI, mean (SD) | 58.2 (10.9) | 59.1 (10.7) | 46.3 (8.7) | 49.6 (10.0) | **<0.001** |
| Education, % (n) |  |  |  |  |  |
| University | 58.1 (265) | 50.5 (53) | 41.7 (55) | 38.6 (22) | **0.004** |
| Vocational training | 30.7 (140) | 37.1 (39) | 41.7 (55) | 38.6 (22) |  |
| High school or less | 11.2 (51) | 12.4 (13) | 16.7 (22) | 22.8 (13) |  |
| Occupation, % (n) |  |  |  |  |  |
| Manager or professional | 64.9 (296) | 58.1 (61) | 53.0 (70) | 57.9 (33) | 0.066 |
| White-collar | 16.7 (76) | 20.0 (21) | 20.5 (27) | 22.8 (13) |  |
| Blue-collar | 10.1 (46) | 10.5 (11) | 20.5 (27) | 12.3 (7) |  |
| Not in labour force | 8.3 (38) | 11.4 (12) | 6.1 (8) | 7.0 (4) |  |
| Serum creatinine (µmol/L), mean (SD) | 72.5 (16.4) | 70.2 (12.1) | 74.6 (16.0) | 72.7 (14.0) | 0.202 |
| Urinary creatinine (mmol/L), median (IQR) | 7.8 (9.1) | 7.0 (8.4) | 11.4 (7.9) | 9.9 (9.1) | **<0.001** |
| Urinary albumin (mg/L) median (IQR) | 3.0 (5.0) | 4.0 (6.0) | 5.0 (7.0) | 4.0 (7.0) | **<0.001** |
| UACR (mg/mmol), median (IQR) | 0.5 (0.7) | 0.7 (0.7) | 0.5 (0.5) | 0.5 (0.7) | **0.015** |
| eGFR (ml/min/1.73m^2^), mean (SD) | 98.0 (11.6) | 98.0 (12.3) | 96.5 (13.1) | 97.8 (11.8) | 0.647 |
| SKD, % (n) | 4.2 (19) | 4.9 (5) | 6.1 (8) | 7.0 (4) | 0.706 |

HLS, healthy lifestyle score; BMI, body mass index; PA, physical activity; DGI: dietary guideline index; SEP, socio-economic position; UACR, urinary albumin-creatinine ratio; eGFR, estimated glomerular filtration rate; SKD, subclinical kidney damage; SD, standard deviation; IQR, interquartile range.

^*^ HLS category was defined as unhealthy with HLS range from 0 to 5, and healthy with HLS range from 6 to 10.

Boldface indicates statistical significance (p<0.05).

| **Supplemental Table 3** The association of childhood HLS as continuous and categorical variables with SKD, UACR, and eGFR in midlife using inverse probability weighting ^*^ | | | | | | | | | |
| --- | --- | --- | --- | --- | --- | --- | --- | --- | --- |
|  |  | **SKD RR (95% CI)** | |  | **UACR (mg/mmol)** | |  | **eGFR (ml/min/1.73m^2^)** | |
|  |  | **RR (95% CI)** | |  | **β 95% CI** | |  | **β 95% CI** | |
|  | **SKD/n (%)** | **Unadjusted** | **Adjusted ^#^** |  | **Unadjusted** | **Adjusted ^#^** |  | **Unadjusted** | **Adjusted ^#^** |
| Childhood HLS | -- | 1.09 (0.84 to 1.41) | 1.15 (0.91 to 1.44) |  | -0.01 (-0.03 to 0.02) | 0 (-0.02 to 0.02) |  | -0.21 (-0.85 to 0.43) | -0.38 (-0.95 to 0.20) |
| Childhood HLS category ^*^ |  |  |  |  |  |  |  |  |  |
| Healthy | 27/580 (4.7) | 1.00 | 1.00 |  | 1.00 | 1.00 |  | 1.00 | 1.00 |
| Unhealthy | 9/160 (5.6) | 0.68 (0.29 to 1.62) | 0.55 (0.24 to 1.27) |  | 0.05 (-0.07 to 0.17) | 0.03 (-0.05 to 0.11) |  | 1.73 (-0.88 to 4.35) | **2.38 (0.08 to 4.68)** |

HLS, healthy lifestyle score; SKD, subclinical kidney damage; UACR, urinary albumin-creatinine ratio; eGFR, estimated glomerular filtration rate; RR, relative risk; CI, confidence interval.

^*^ Age, sex, and school type at baseline were used to impute missing data; socio-economic position, BMI category, waist circumference z-score, waist to height ratio, fitness [sit and reach (cm), sit-ups (number), standing long jump (cm), time for 1.6 km run (minutes)], school enjoyment, school assessed scholastic ability, self-reported health status, smoking experimentation, and passive smoking at baseline were used to determine the weights.

^#^ childhood HLS category was defined as unhealthy with HLS range from 0 to 5, and healthy with HLS range from 6 to 10.

^ƚ^ adjusted for childhood age, sex, socio-economic position in childhood, education, occupation in midlife, and the duration of follow-up.

| **Supplemental Table 4** The association of HLS category ^*^ from childhood to adulthood with SKD, UACR, and eGFR in midlife using inverse probability weighting ^#^ | | | | |
| --- | --- | --- | --- | --- |
|  | **SKD/n (%)** | **SKD**  **RR (95% CI)** | **UACR (mg/mmol)**  **β (95% CI)** | **eGFR (ml/min/1.73m^2^)**  **β (95% CI)** |
| Persistently healthy | 19/449 (4.2) | 1.00 | 1.00 | 1.00 |
| Improving | 5/103 (4.9) | 0.81 (0.30 to 2.19) | 0.05 (-0.03 to 0.14) | 1.10 (-1.28 to 3.48) |
| Worsening | 8/131 (6.1) | **2.32 (1.01 to 5.31)** | 0 (-0.07 to 0.08) | -1.28 (-3.65 to 1.09) |
| Persistently unhealthy | 4/57 (7.0) | 0.77 (0.23 to 2.52) | 0.01 (-0.11 to 0.13) | 3.21 (-0.08 to 6.50) |

UACR, urinary albumin-creatinine ratio; eGFR, estimated glomerular filtration rate; SKD, subclinical kidney damage; RR, relative risk; CI, confidence interval.

^*^ HLS category was defined as unhealthy with HLS range from 0 to 5, and healthy with HLS range from 6 to 10.

^#^ Age, sex, and school type at baseline were used to impute missing data; socio-economic position, BMI category, waist circumference z-score, waist to height ratio, fitness [sit and reach (cm), sit-ups (number), standing long jump (cm), time for 1.6 km run (minutes)], school enjoyment, school assessed scholastic ability, self-reported health status, smoking experimentation, and passive smoking at baseline were used to determine the weights.

Note: adjusted for childhood age, sex, socio-economic position in childhood, education, occupation in midlife and the duration of follow-up.
